# Implementation of optically simulated luminescent dosimeter for quality control of gamma ray dose of an accelerator‐based neutron source

**DOI:** 10.1002/acm2.14493

**Published:** 2024-08-27

**Authors:** Naonori Hu, Taiki Nakamura, Ryusuke Kataura, Keita Suga, Tetsuya Mukawa, Kazuhiko Akita, Akinori Sasaki, Mai Nojiri, Nishiki Matsubayashi, Takushi Takata, Hiroki Tanaka, Keiji Nihei, Koji Ono

**Affiliations:** ^1^ Osaka Medical and Pharmaceutical University Kansai BNCT Medical Center Osaka Japan; ^2^ Institute for Integrated Radiation and Nuclear Science Kyoto University Osaka Japan; ^3^ Sumitomo Heavy Industries, Ltd. Tokyo Japan; ^4^ Graduate School of Engineering Kyoto University Kyoto Japan; ^5^ Osaka Medical and Pharmaceutical University Hospital Department of Radiation Oncology Osaka Japan

**Keywords:** accelerator neutron source, BNCT, Monte Carlo simulation, OSLD, QA

## Abstract

**Background:**

Neutron beams utilized for performing BNCT are composed of a mixture of neutrons and gamma rays. Although much of the dose delivered to the cancer cells comes from the high LET particles produced by the boron neutron capture reaction, the dose delivered to the healthy tissues from unwanted gamma rays cannot be ignored. With the increase in the number of accelerators for BNCT, a detector system that is capable of measuring gamma ray dose in a mixed neutron/gamma irradiation field is crucial. Currently, BeO TLDs encased in quartz glass are used to measure gamma ray dose in a BNCT irradiation field. However, this type of TLD is no longer commercially available. A replacement dosimetry system is required to perform the recommended ongoing quality assurance of gamma ray measurement for a clinical BNCT system.

**Purpose:**

The purpose of this study is to evaluate the characteristics of a BeO OSLD detector system under a mixed neutron and gamma ray irradiation field and to assess the suitability of the system for routine quality assurance measurements of an accelerator‐based BNCT facility.

**Methods:**

The myOSLD system by RadPro International GmbH was evaluated using the accelerator‐based neutron source designed for clinical BNCT (NeuCure BNCT system). The readout constancy, linearity, dose rate effect, and fading effect of the OSLD were evaluated. Free‐in‐air and water phantom measurements were performed and compared with the TLD results and Monte Carlo simulation results. The PHITS Monte Carlo code was used for this study.

**Results:**

The readout constancy was found to be stable over a month‐long period and similar to the TLD results. The OSLD readout signal was found to be linear, with a high coefficient of determination (*R* ^2^ ≥ 0.999) up to a proton charge of 3.6 C. There was no significant signal fading or dose rate dependency. The central axis depth dose and off‐axis dose profile measurements agreed with both the TLD and Monte Carlo simulation results, within one standard deviation.

**Conclusion:**

The myOSLD system was characterized using an accelerator system designed for clinical BNCT. The experimental measurements confirmed the OSLD achieved similar, if not superior to, the currently utilized dosimetry system for routine QA of an accelerator‐based BNCT system. The OSLD system would be a suitable replacement for the current TLD system for performing routine QA of gamma ray dose measurement in a BNCT irradiation field.

## 1 INTRODUCTION

Boron neutron capture therapy (BNCT) utilizes the high linear energy transfer (LET) particles that are emitted when a thermal neutron is captured by a ^10^B atom.^1^ These particles travel a short range in tissue, depositing practically all their energy in the cell where the reaction took place, making it an ideal cancer treatment, assuming selective ^10^B accumulation in the cancer cell can be achieved. In the past, neutrons for BNCT were generated using a nuclear reactor. Nowadays, the trend is shifting toward an accelerator to generate neutrons for BNCT. The BNCT irradiation field consists of a mixture of both neutrons and gamma rays. Accurate determination of both neutron and gamma ray dose rate is essential to calculate the dose delivered to the patient.

In a typical BNCT irradiation field, gamma rays are generated from the reaction between thermal neutrons and hydrogen atoms inside the human body, ^1^H(n,γ)^2^H. Additionally, as neutrons traverse through the beam shaping assembly, they activate various components, which also produce gamma rays. The dose deposited from the summation of these gamma rays (originating from the accelerator components and the patient itself) cannot be ignored when calculating the total dose deposited to the patient. For example, the cyclotron‐based accelerator system installed at the Kansai BNCT Medical Center (NeuCure BNCT system, hereafter NeuCure), approximately 20% of the total dose delivered to the normal tissue results from the gamma ray contribution.^2^

A detector system to measure gamma rays in a mixed radiation field is necessary. In conventional radiotherapy, ionization chambers are often used to determine the absorbed dose inside a medium due to its high precision and accuracy. Few authors have investigated the use of an ionization chamber for dose measurement of a BNCT irradiation field using a combination of different gas mixtures inside the cavity volume.^3^, ^4^, ^5^ However, problems arise from the activation of the gas inside the cavity and depending on the local regulations, handling of activated gas in a hospital environment can be a difficult task.

In the past, beryllium oxide thermoluminescent dosimeters (TLDs) encased in a special quartz glass, which is insensitive to thermal neutrons, have been utilized for gamma ray detection of reactor‐based BNCT irradiation field^6^, ^7^ and an accelerator‐based BNCT irradiation field^8^, ^9^, ^10^ and is currently routinely used for quality control tests at clinical BNCT centers.^10^, ^11^, ^12^ However, one major disadvantage is that they are no longer manufactured. One of the reasons is because it is in powdered form and can easily be dispersed when the glass casing is damaged, and given that BeO is highly toxic, having it in powdered form is not ideal.^13^ Therefore, a dosimeter to replace the TLD that is currently used in the clinic for gamma ray detection of a BNCT irradiation field is required.

The use of an optically simulated luminescent dosimeter (OSLD) for radiotherapy application is increasing and the American Association of Physicists in Medicine (AAPM) presented a method for point dose measurements in the Task Group 191 report for TLD and OSLD.^12^, ^14^ There are various materials available and one that is widely used is carbon‐doped aluminium oxide (Al_2_O_3_:C). However, both low and high energy neutrons react with aluminium (^27^Al(n,α)^24^Na, ^27^Al(n,γ)^28^Al),^15^, ^16^, ^17^ which have been used to characterize neutron spectrum and evaluate the dose from high energy neutrons,^8^, ^18^ but for the measurement of pure gamma rays, an alternative material is desirable. Although the study by Santos et al. has shown that the photon and fast neutron component can be determined separately using an Al_2_O_3_ TLD, two measurements need to be performed with a time delay between each reading, which is not suitable for a busy clinical environment.

Recent studies on BeO OSLD have shown that it can be a potential alternative to Al_2_O_3_:C and may be preferable for x‐ray radiotherapy applications due to the low energy dependency, linear response at high doses, and effective atomic number being close to tissue.^19^, ^20^, ^21^, ^22^, ^23^ Matsubayashi et al. investigated a ceramic BeO OSLD for measurement of gamma ray dose in a reactor‐based BNCT irradiation field.^24^ They determined the thermal neutron sensitivity using the Kyoto University Reactor and found it to be less than the powdered BeO TLD encased in quartz glass.

In this work, the BeO OSLD will be characterized using a clinical accelerator‐based neutron source designed for BNCT, and its suitability and potential application for routine quality control measurement of gamma ray doses will be assessed.

## 2 METHOD

**FIGURE 1:**
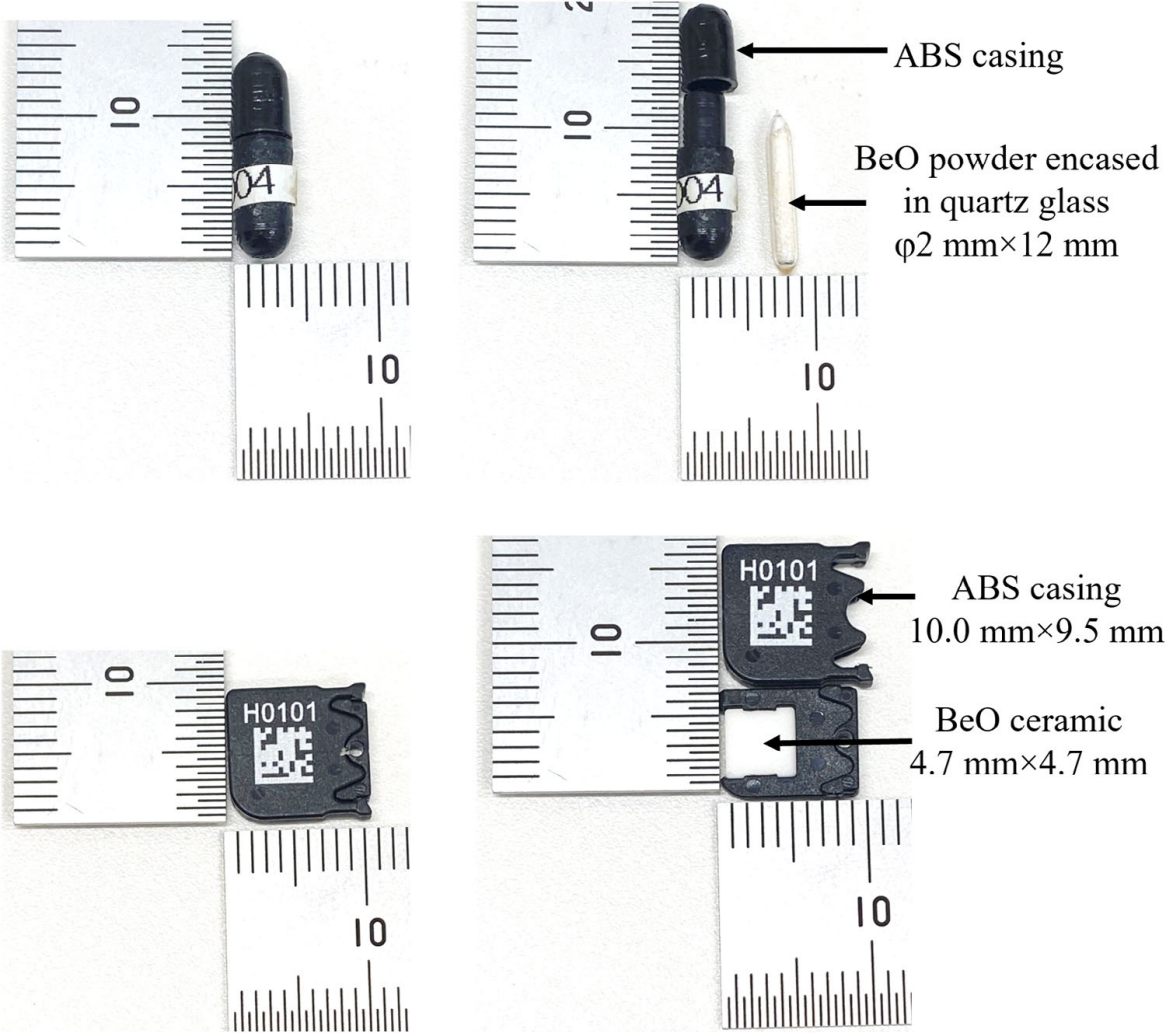
Image of the BeO OSL chip and BeO powdered TLD used in this study.

The myOSLD system (RadPro International GmbH) was used for this study. The measurement chamber contains a super bi‐alkaline cathode photomultiplier tube (Hamamatsu) with 460 nm wavelength light for stimulation and erasure of the OSL chip. The chip is a BeO element with dimensions of 4.7 × 4.7 × 0.5 mm, encased in a sheath made from Acrylnitril‐Butadien‐Styrol‐Copolymere (ABS) with dimensions of 10.0 × 9.5 × 2.0 mm (see Figure 1). Low‐dose and high‐dose mode is available with the system. For this study, the high‐dose mode was selected. With the high‐dose mode, the OSL chips are pre‐irradiated with 6 MeV electron beam to a dose of 10 Gy to reach stability in the sensitivity.^25^ This process is performed by the vendor.

For the TLD measurements, powdered BeO encased in quartz glass (Panasonic UD‐170LS) was used. This device consisted of Na‐doped BeO grains encapsulated in a 2 mm diameter, 12 mm height quartz glass tube. The Panasonic UD‐5120PGL TLD reader was used to measure the signal of the TLD. A comparison of the OSLD and TLD used in this study is shown in Table 1.

**TABLE 1:** Properties of the TLD and OSLD used in this study.

Detector	Material composition	State	Outer casing material	Geometry
TLD	BeO	Powder	ABS + Quartz glass	Cylinder (height: 12 mm, diameter: 2 mm)
OSLD		Solid	ABS	Rectangle (4.7 mm × 4.7 mm × 0.5 mm)

Both dosimeters were calibrated using the ^60^Co source (414 TBq as of February 2008) at Kyoto University Institute for Integrated Radiation and Nuclear Science. Although the dominant photon energy in a BNCT irradiation field is 2.2 MeV, Ando et al. have shown the beam quality conversion factor can be treated as unity for high energy photon beams and it is possible to perform accurate dosimetry even when the calibration coefficient determined for ^60^Co source is applied.^28^ Each dosimeter was placed in the center of a 20 × 20 × 20 cm cubic phantom filled with water. A secondary standard ionization chamber (source of traceability: Japan Calibration Service System) was used to determine the absorbed dose at the location of the dosimeter using the following relationship:

(1) $$D=N_{D,w,Co}\cdot M\cdot \left(\frac{T\cdot P_{0}}{T_{0}\cdot P}\right)$$

where *N_D,w,Co_* is the calibration coefficient in terms of absorbed dose to water (Gy/signal), *M* is the chamber signal, *T* and *P* are chamber air pressure and temperature, respectively, at the time of measurement, and *T_0_* (22°C) and *P_0_* (101 kPa) are the normal conditions used in the standards laboratory. The water phantom was placed 150 cm away from the source, such that the dose rate at the location of the dosimeter/ionization chamber would be approximately equal to the maximum gamma ray dose rate inside a patient during BNCT (∼1 × 10^−3 ^Gy/s, for the conditions stated above). The sensitivity of each element (*ε*) was determined by subtracting the specific zero‐signal (OSL signal at zero dose, *S_0_*) from the OSL signal (*S_cal_*) and dividing it by the known dose (*D_cal_*), as shown below:

(2) $$\varepsilon =\left(S_{Cal}-S_{0}\right)/D_{Cal}$$

**FIGURE 2:**
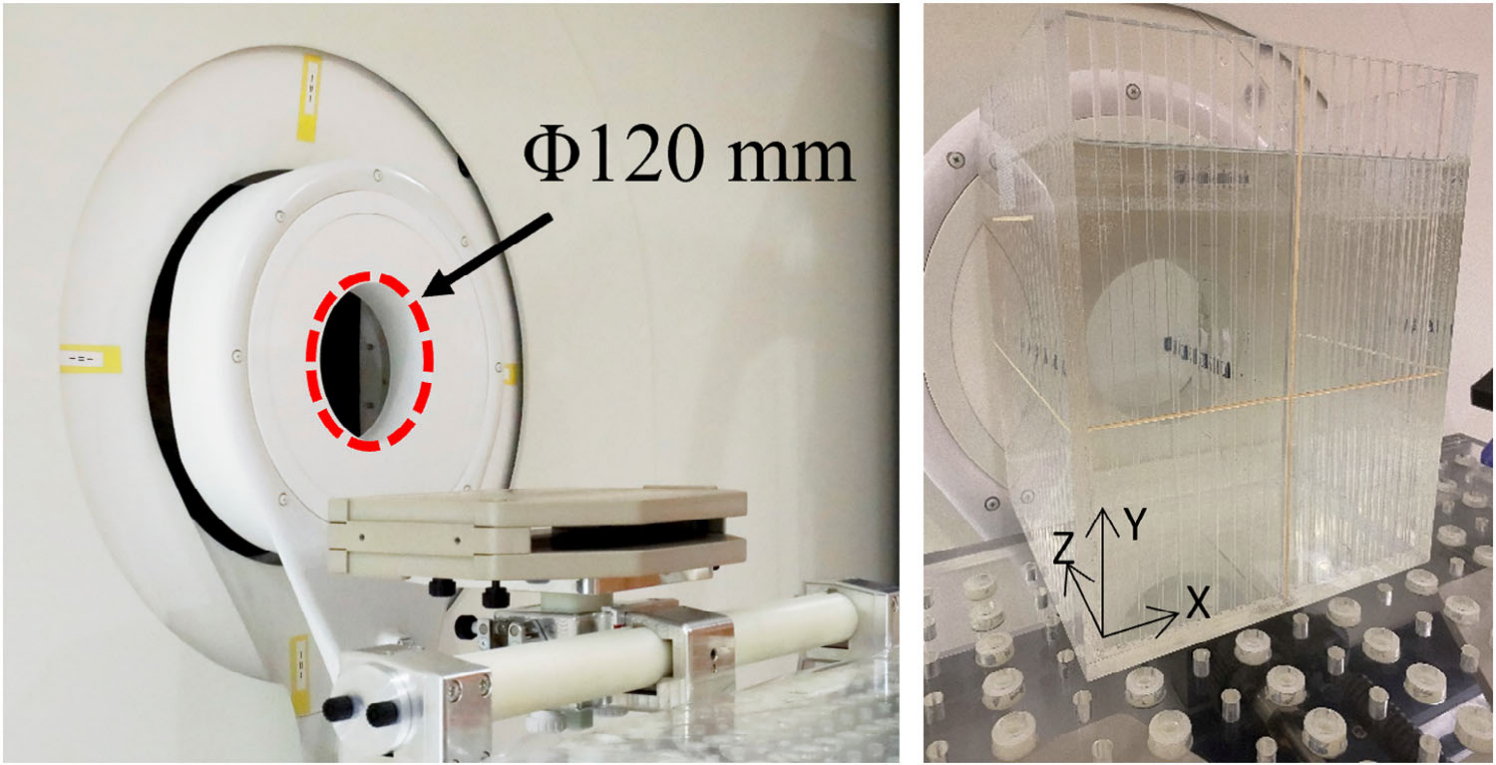
(Left) 120 mm diameter circular aperture of the NeuCure BNCT system. (Right) Water phantom used during the experiment.

**FIGURE 3:**
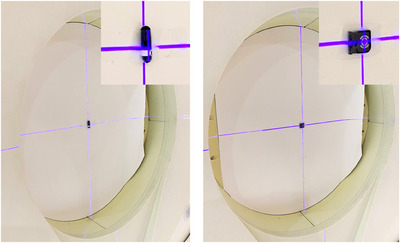
(Left) TLD placed free‐in‐air on the wall. (Right) OSLD placed free‐in‐air on the wall.

Once the calibration factor and the sensitivity of each individual element were determined, experimental measurements in a mixed neutron‐gamma ray field were performed using the accelerator‐based neutron source at the Kansai BNCT Medical Center (NeuCure).^26^ The reference field size used was 120 mm diameter circular aperture (Figure 2). Unlike clinical linear accelerators used in conventional radiotherapy, where the beam output is set by the number of monitor units delivered to the ionization chamber (which is equal to the absorbed dose in water under reference conditions), there is no beam monitor system for a BNCT irradiation system that can measure the total dose (a combination of neutron dose and gamma ray dose) in real‐time. Therefore, the total number of proton charge (integrated current) delivered to the target is used to control the beam output in BNCT.^27^ Measurements were performed both free‐in‐air and inside a water phantom (height: 280 mm, length: 210 mm, width: 210 mm). For the free‐in‐air measurement, the dosimeters were placed on the beam exit wall (Figure 3). A thin sheet of paper was placed between the wall and the dosimeter to prevent direct contact with lead (wall).

### 2.1 Fading effect

The change in the OSLD signal post‐irradiation was measured by placing two OSL chips (side‐by‐side) on the beam exit wall, free‐in‐air, and delivering a proton charge of 0.1 C. One chip was measured 10 min after irradiation and the other chip was measured on different days after irradiation (1, 2, 3, 4, 5, 6, 8, and 10 days). The measurements were repeated three times. The sensitivity of each OSL chip was accounted for during the readout.

### 2.2 Dose rate dependency

The effect of the dose rate on the OSLD was investigated by changing the proton current of the accelerator from 200  to 1000 µA, in increments of 200 µA. The OSL chip was placed at 2 cm depth inside the water phantom. Measurements were repeated three times.

### 2.3 Dose response (linearity)

The dose response of the OSLD was investigated by delivering different amounts of proton charge and measuring the signal delivered to the chip. Proton charges of 0.1, 0.3, 0.6, 1.2, 2.4, and 3.6 C were delivered to the OSLD placed at 2 cm inside the water phantom. This equated to a dose of 0.1, 0.3, 0.7, 1.4, 2.7, and 4.1 Gy. Measurements were repeated three times.

### 2.4 Comparison with TLD

#### 2.4.1 Daily measurement free in air (Daily QA)

Measurements were performed daily over a 1‐month period to evaluate the reproducibility of the OSLD. A single OSL chip was placed at the center of the irradiation field at the surface of the beam exit wall. A proton charge of 0.1 C was delivered. The measurements were repeated twice each day. The standard deviation (SD) and the coefficient of variation (CV) were calculated. The measurements were compared with TLD results.

(3) $$SD\;=\sqrt{\frac{\sum {\left(x_{i}-\mu \right)}^{2}}{N}}\;\;$$

(4) $$CV\;=\frac{SD}{\mu }\;\;$$

where *x_i_* is each value from the population size *N* and *μ* is the population mean.

#### 2.4.2 Weekly measurement inside a water phantom (Weekly QA)

Weekly QA measurements were performed using a water phantom. Measurements were performed by placing the OSL chip at the surface of the water phantom and at a depth of 2 and 6 cm inside the phantom along the central beam axis. A proton charge of 0.3 C was delivered, and the dose rate (Gy/s) at each depth was calculated and compared with the TLD measurements. Two sets of measurements were performed for each dosimeter (TLD, OSLD).

#### 2.4.3 Monthly measurement inside a water phantom (Monthly QA)

The central axis depth dose curve was measured by placing the OSL chip along the central beam axis at 1 cm intervals up to a depth of 12 cm. The off‐axis dose profile at depths of 2 and 6 cm was measured at 2 cm intervals. All measurements were repeated three times. The measurements were compared with the TLD measurements.

**FIGURE 4:**
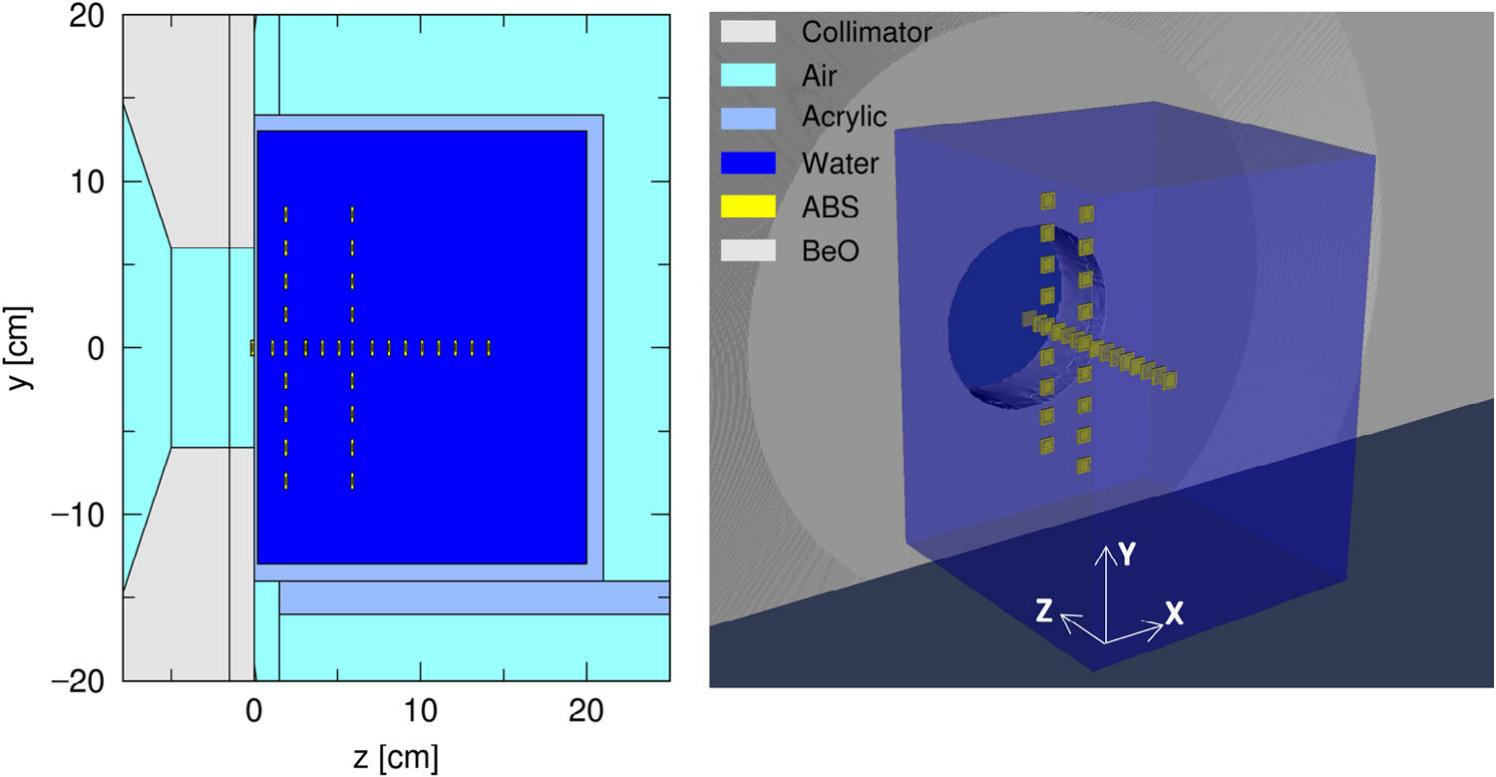
(Left) 2D geometry of the experimental set up modeled using PHITS. (Right) 3D representation of the experimental set up.

In addition to experimental measurements, Monte Carlo simulation was performed using Particle and Heavy Ion Transport code System (PHITS^29^), and the results were compared with experimental measurements. The simulation geometry and parameters of the NeuCure have been evaluated in a previous study.^30^ The monthly QA set up was modeled (water phantom with BeO chips placed along the beam central axis and off‐axis at depths of 2 and 6 cm, Figure 4) and the T‐deposit tally using the EGS5 (Electron Gamma Shower) algorithm was used to simulate the electron transport.^31^

## 3 RESULTS

### 3.1 Fading effect

**FIGURE 5:**
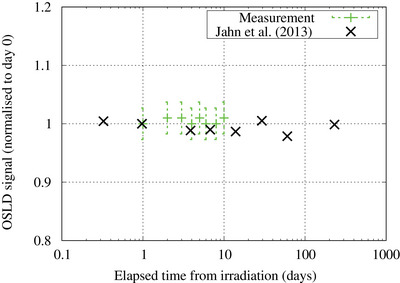
The fading effect of the OSL chip. Error bar represents the standard deviation of the readings.

The change in the OSLD signal readout with time is shown in Figure 5. The variation in the signal was found to be within the readout accuracy of the OSLD system, and no significant fading in the signal was observed up to 10 days after exposure. Compared with a previous study by Jahn et al., a similar trend was obtained.^25^

### 3.2 Dose rate dependency

**FIGURE 6:**
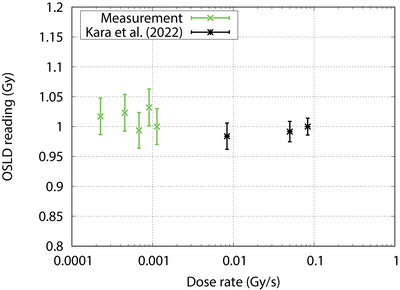
Dose rate response curve of the OSLD. Error bar represents the standard deviation of the readings.

The dose rate response of the OSLD is shown in Figure 6. No significant change in the OSLD reading was observed with the changes in the dose rate of the accelerator (i.e., dose rate independent). Compared with a previous study performed by Kara et al., where they used a high energy photon beam with a dose rate of 50, 300, and 500 MU/min, a similar trend was obtained.^32^

### 3.3 Dose response (linearity)

**FIGURE 7:**
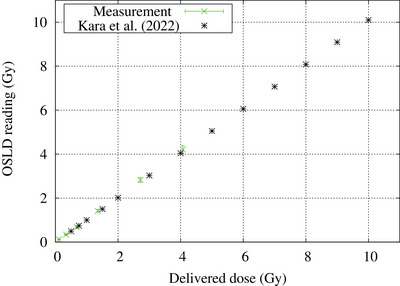
Dose‐response curve of the OSL chip.

The dose response of the OSLD is shown in Figure 7. A linear relationship (R‐squared greater than or equal to 0.999) between the OSLD reading and the delivered dose (ranging from 0.1 to 4.1 Gy) was observed. Compared with a previous study by Kara et al., similar results were obtained.^32^

### 3.4 Comparison with TLD

#### 3.4.1 Daily measurement free in air (Daily QA)

**FIGURE 8:**
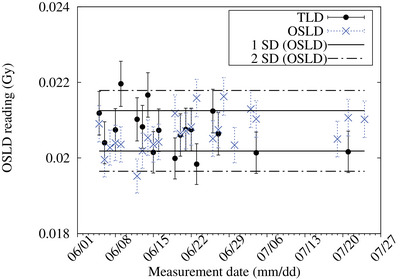
Daily free in air measurement of both OSLD and TLD were conducted over a one‐month period. The accelerator was under maintenance between July 7th and July 18th; hence, no measurements were performed during this period. Error bars represent the standard deviation of the readings.

The average dose was measured to be 0.0207 Gy for both OSLD and TLD for a proton charge of 0.1 C. The SD and CV were 0.0005 Gy and 2.6%, respectively, for the OSLD and 0.0006 Gy and 2.7%, respectively, for the TLD (Figure 8).

#### 3.4.2 Weekly measurement inside a water phantom (Weekly QA)

The weekly QA measurement results are shown in Table 2. The measurement at 2 and 6 cm depths showed similar results between the OSLD and TLD. However, at the surface of the phantom, the OSLD measurement was higher than the TLD measurement.

**TABLE 2:** Measurements were taken at the surface, 2 , and 6 cm depths inside a water phantom using both OSLD and TLD for routine weekly QA.

Depth (cm)	Dose rate (×10^−3^ Gy/s)	
	OSLD ± 1 SD (CV%)	TLD ± 1 SD (CV%)
0	0.87 ± 0.02 (1.8)	0.74 ± 0.02 (2.6)
2	1.12 ± 0.03 (2.2)	1.13 ± 0.04 (3.5)
6	0.82 ± 0.03 (3.1)	0.79 ± 0.03 (3.4)

The measurements were repeated three times at each depth.

#### 3.4.3 Monthly measurement inside a water phantom (Monthly QA)

**FIGURE 9:**
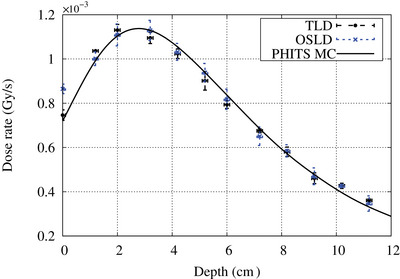
Central axis depth dose curves measured with both OSLD and TLD inside a water phantom. The solid line indicates the PHITS Monte Carlo simulation result. Error bar represents the standard deviation of the readings.

**FIGURE 10:**
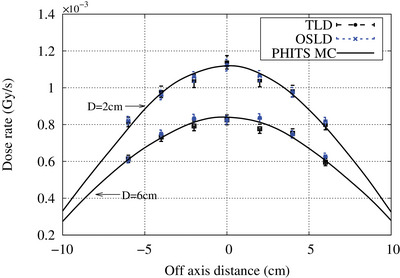
Lateral dose profile measured with both OSLD and TLD at a depth of 2 and 6 cm inside a water phantom. The solid line indicates the PHITS Monte Carlo simulation result. Error bar represents the standard deviation of the readings.

The central axis depth dose curve and the off‐axis depth dose curve at a depth of 2 and 6 cm are shown in Figures 9 and 10, respectively. Except for the measurements at the surface, both the OSLD and TLD results closely matched each other (within experimental uncertainty) and closely matched with the PHITS Monte Carlo simulation results.

## 4 DISCUSSION

OSLDs for x‐ray therapy dosimetry have been studied in the past by several other authors^21^, ^22^, ^23^, ^33^ and a report by the AAPM (TG 191) summarizes the clinical use of both TLDs and OSLDs. Despite extensive studies being reported on use of OSLDs, only a few studies show the use of OSLD in a neutron irradiation field,^24^, ^34^ and there are no reports of BeO OSLDs being utilized for routine QA/QC measurement of an accelerator‐based BNCT irradiation system. This study shows the first application of the myOSLD system for measurement of gamma ray dose of an accelerator‐based BNCT irradiation field using the NeuCure BNCT system at the Kansai BNCT Medical Center.

In a study performed by Jahn et al., the signal of the BeO OSLDs decreased by approximately 20% in the first 5 min.^25^ After 5 min, the signal remained constant and long‐term fading was found to be negligible under environmental conditions. At the Kansai BNCT Medical Center, to minimize the staff's exposure to radiation, there is a waiting time of at least 5 min before the staff can enter the room after irradiation. Therefore, the aforementioned limitation of the BeO OSLD will not be an issue, and the readout can be performed immediately after the OSLDs are taken out of the irradiation room. This will improve the efficiency of routine QA, as the current method (TLD measurement) requires 60 min of waiting time before the readout can be performed. No significant change in the signal was observed 10 days post neutron irradiation. This result is consistent with the results by Abusaid et al., and Jursinic, where they found no significant change in the OSLD signal over a 2 and 24 h post irradiation readout, respectively,^23^, ^35^ meaning radioactivation of the dosimeter from neutron irradiation has little effect on the OSLD signal over time. This property makes it advantageous for clinical institutions where readouts cannot be performed immediately after treatment due to busy workload. Furthermore, it may be used as a postal audit system, where it may take a few days for the OSL chips to be returned to the readout facility.

The dose rate response of the OSLD was evaluated and no significant difference in the OSLD signal was observed for a dose rate ranging from 0.2 to 1.1×10^−3 ^Gy/s. The OSLD dose response between 0.1 and 4.1 Gy was found to be linear with a coefficient of determination of greater than or equal to 0.999. These results are also consistent with the results by Kara et al. and Abusaid et al.^21^, ^23^ These results show there are no large differences in the OSLD signal when used in a mixed neutron‐gamma ray irradiation field and a high energy photon beam.

Currently, for clinical BNCT irradiation (performed according to the rules set by the Japanese government surrounding the safety of the boron drug infusion time), the maximum irradiation time is limited to 1 h. For a proton beam current of 1 mA with a 1‐h irradiation time, the total proton charge is calculated to be 3.6 C. For routine QA, a proton charge of 0.1–0.3 C (daily and weekly QA, respectively) is usually delivered. During this study, the maximum accumulated dose for a single OSL chip was approximately 15 Gy. The calibration factor of each chip was unaltered throughout the study. An OSL chip placed at a depth of 2 cm inside the water phantom resulted in a reading of approximately 0.3 Gy (for a proton charge of 0.3 C). This means that a single chip can be used for weekly QA (i.e., a single measurement of 0.3 Gy each week) at least for a year without the need for calibration, as the OSLD signal remained constant up to a cumulative dose of 15 Gy.

The results of the daily, weekly, and monthly QA tests were compared with the TLD measurements. The results showed that the OSLD measurements were similar to the TLD measurements. A slight improvement in reproducibility was observed with the OSLD measurements in comparison to the TLD measurements. A discrepancy in the dose at the surface of the phantom was observed. The cause of this discrepancy is unknown. One possible reason could be the difference in the geometry of the two detectors. The OSLD has a greater surface area, which may make it more sensitive in regions where changes in the thermal neutron flux are steep (i.e., the surface of the water phantom). Further investigation is required to confirm this hypothesis. Another possible explanation could be due to the recoil proton events caused by the fast neutrons within the neutron irradiation field. Figure 11b in the appendix shows the simulated dose distribution along the central beam axis inside a water phantom. The fast neutron flux at the surface of the phantom is high and decreases rapidly as a function of depth. Furthermore, the outer casing of the OSLD is made from ABS, which contains small amounts of hydrogen, which may lead to the production of recoil protons (^1^H(n,n’)p). The recoil protons produced inside the phantom (i.e., water) are unavoidable, but as for the outer casing, by using a material that does not contain any hydrogen (e.g., polytetrafluoroethylene), these events may be reduced.

## 5 CONCLUSION

The application of the myOSLD system for gamma ray measurement in an accelerator‐based BNCT irradiation field was evaluated. The linearity and repeatability of the system were found to be the same, if not superior, in comparison with the current TLD system used for routine QA of the accelerator BNCT system at the Kansai BNCT Medical Center. The myOSLD system would be a suitable replacement device for routine QA measurement of an accelerator‐based BNCT system and improve the efficiency of the routine QA.

## AUTHOR CONTRIBUTIONS

Conceptualization and methodology, Naonori Hu and Taiki Nakamura; Data analysis and process, Ryusuke Kataura, Keita Suga, Tetsuya Mukawa, Akinori Sasaki, and Mai Nojiri; Resources, Kazuhiko Akita, Nishiki Matsubayashi, and Takushi Takata; Writing, Naonori Hu; Supervision, Hiroki Tanaka, Keiji Nihei, Koji Ono. All authors have read and agreed to the published version of the manuscript.

## CONFLICT OF INTEREST STATEMENT

The authors declare the following financial interests/personal relationships which may be considered as potential competing interests: Naonori Hu and Hiroki Tanaka partially received research funding from Sumitomo Heavy Industries, Ltd. Taiki Nakamura, Ryusuke Kataura, Keita Suga, and Tetsuya Mukawa are employees of Sumitomo Heavy Industries, Ltd. Other authors have no conflicts of interest associated with this manuscript.
